# Daptomycin-Related Rhabdomyolysis Complicated by Severe Hyperkalemia and Acute Kidney Injury

**DOI:** 10.7759/cureus.29764

**Published:** 2022-09-29

**Authors:** Yoshito Nishimura

**Affiliations:** 1 Internal Medicine, University of Hawaii, Honolulu, USA

**Keywords:** acute kidney injury, hyperkalemia, statin, daptomycin, rhabdomyolysis

## Abstract

We describe a case of a 65-year-old man who presented with progressive generalized weakness. He was started on daptomycin for methicillin-resistant Staphylococcus aureus (MRSA) right fifth metatarsal osteomyelitis. Laboratory testing revealed severe hyperkalemia of 7.5 mEq/L (normal range: 3.3-5.1) and acute kidney injury (AKI) stage 1 associated with a substantial elevation in serum creatine kinase (CK). He had been on a high-intensity statin for years, and daptomycin was switched to linezolid, and statin was held as daptomycin-related rhabdomyolysis was suspected. Our case highlights the importance of seeking alternatives to daptomycin for patients on chronic statins.

## Introduction

Daptomycin is a lipopeptide antibiotic often used for the treatment of methicillin-resistant Staphylococcus aureus (MRSA). Despite its clinical utility, there have been a few reports of rhabdomyolysis related to daptomycin use [1,2]. In this report, we present a case of rhabdomyolysis, severe hyperkalemia, and acute kidney injury (AKI) that occurred 10 days after the co-administration of daptomycin 480 mg and atorvastatin 80 mg daily. Given the well-known fact that statins, and lipophilic statins such as atorvastatin, fluvastatin, and simvastatin, in particular, could be myotoxic, patients on statin therapy should be rigorously evaluated before the initiation of daptomycin to see if there are any alternatives to the antibiotic.

## Case presentation

A 65-year-old man with a history of insulin-dependent diabetes mellitus type 2 (IDDM2), hypertension, chronic kidney disease (CKD) stage G3aA3 (baseline creatinine of 1.0 mg/dL), and chronic MRSA osteomyelitis of the right foot presented to the emergency department (ED) with a two-day history of progressive generalized weakness. He had been admitted to the hospital two weeks prior for MRSA osteomyelitis of the right foot. He had been discharged home with daily intravenous daptomycin 6 mg/kg for 10 days prior to the ED visit. His medical history was significant for chronic MRSA osteomyelitis of the right foot for which he had undergone fourth-toe amputation two months ago. His medications other than daptomycin included atorvastatin 80 mg daily. Upon presentation, he was hypertensive with a blood pressure of 217/68 mmHg and a pulse rate of 62 per minute. His BMI was 24.9 kg/m^2^. He was alert and afebrile. There was pitting edema of bilateral lower extremities. Also, an ulcerative lesion on the right foot without surrounding erythema or exudate was noted. On neurological examination, deep tendon reflexes were grossly intact. Muscle strength was 4/5 throughout. No muscle wasting or fasciculations were noted. The results of laboratory tests upon presentation are summarized in Table 1. On admission, potassium was 7.5 mEq/L and creatinine was 1.8 mg/dL. No cast was present in urinalysis. These findings raised concerns about rhabdomyolysis in the setting of recent daptomycin initiation and prompted us to check his serum total creatine kinase (CK), which was 19,460 IU/L (reference range: 39-308). Of note, serum CK nine days prior to admission before the initiation of daptomycin had been normal at 55 IU/L. An electrocardiogram showed atrial fibrillation with a slow ventricular response at a rate of 42 beats per minute without peaked T waves, which was believed to be related to hyperkalemia. CT of the abdomen to pelvis without contrast showed no hepatic abscess, choledocholithiasis, or urinary tract obstruction.

**Table 1:** Laboratory values on admission ALP: alkaline phosphatase; ALT: alanine aminotransferase; AST: aspartate aminotransferase; BUN: blood urea nitrogen; eGFR: estimated glomerular filtration rate; LDH: lactate dehydrogenase; TSH: thyroid-stimulating hormone; WBC: white blood cells

Parameter	Value	(Reference range)/units
WBC	12.5	(3.8-10.8 x 10^3^)/µL
Hemoglobin	8.7	(13.7-17.5) g/dL
Platelet	393	(151-424 x 10^3^)/µL
Sodium	125	(133-145) mEq/L
Potassium	7.5	(3.3-5.1) mEq/L
Chloride	93	(95-108) mEq/L
Calcium	8.4	(8.3-10.5) mg/dL
Phosphorus	4.5	(2.5-4.5) mg/dL
BUN	43	(6-23) mg/dL
Creatinine	1.8	(0.6-1.4) mg/dL
eGFR	45	(>90) mL/min/1.73 m^2^
AST	525	(0-40) IU/L
ALT	132	(0-41) IU/L
ALP	97	(35-129) IU/L
Total bilirubin	0.4	(0-1.2) mg/dL
Total protein	7.0	(6.4-8.3) g/dL
Albumin	3.3	(3.5-5.2) g/dL
LDH	718	(135-250) IU/L
Creatine kinase	19460	(39-308) IU/L
Troponin T generation 5	129	(<19) ng/L
TSH	0.19	(0.27-4.20) µIU/mL
Free T4	1.6	(0.9-2.1) ng/dL
Hemoglobin A1c	8.3	(<5.7)%
Urine analysis		
Appearance	Clear	
Gravity	1.005	(1.005-1.030)
pH	6.5	(5.0-7.5)
Protein	1+	
Glucose	Negative	
Ketones	Negative	
Blood	Large	
Red blood cells	0-2/hpf	(0-2)/hpf
Urine eosinophils	Negative	

Given his severe hyperkalemia, the patient was treated with calcium carbonate, intravenous insulin, and glucose at first along with furosemide. For rhabdomyolysis and AKI, he was managed with aggressive intravenous fluids therapy with normal saline. Hyperkalemia was corrected appropriately, and hypertension also resolved spontaneously without antihypertensive medications. Atorvastatin and daptomycin were discontinued. Oral linezolid was started in place of daptomycin for MRSA osteomyelitis. Serum CK level peaked at 30090 IU/L on hospital day two and then trended down to 4100 IU/L on hospital day six (Table 2). The patient was discharged home on hospital day six with oral linezolid.

**Table 2:** Trends of creatine kinase, creatinine, and liver function tests ALT: alanine aminotransferase; AST: aspartate aminotransferase; eGFR: estimated glomerular filtration rate

Parameter	Day 1	Day 2	Day 3	Day 4	Day 5	Day 6	(Reference range)/units
Creatine kinase	19460	30090	26010	20153	10516	4100	(39-308) IU/L
Creatinine	1.8	1.7	1.7	1.7	1.4	1.3	(0.6-1.4) mg/dL
eGFR	45	48	48	48	61	66	(>90) mL/min/1.73 m^2^
AST	525	1101	1137	1104	795	447	(0-40) IU/L
ALT	132	259	318	345	332	285	(0-41) IU/L

## Discussion

Daptomycin is a relatively new class of antibiotics categorized as a cyclic lipopeptide. It has bactericidal activity against Gram-positive pathogens and is commonly used for complicated skin and soft tissue infection (SSTI) caused by MRSA or vancomycin-resistant S. aureus infection. Because it can be administered as a once-daily infusion, daptomycin is sometimes used as an alternative to other anti-MRSA agents for long-term outpatient antibiotic therapy [3]. Despite its clinical usefulness, daptomycin can cause skeletal muscle toxicity as the main side effect. A randomized trial reported that 2.1% (11/534) of the patients who received daptomycin for SSTI had an increase in CK. At the same time, severe myopathy was considered to be rare, with only 0.2% of patients on daptomycin noted to have an elevated CK >10 times the upper limit of normal. While the exact mechanism of myotoxicity with daptomycin has been unclear to date, it has been hypothesized that daptomycin might cause pore-like formation on rhabdomyocytes intertwined with cellular membranous lipids. At a high dose, the pore-like formation could induce rhabdomyocytes depolarization leading to cellular lysis [4]. Several cases of daptomycin-induced severe rhabdomyolysis have been described in the literature [4,5]. In most cases, CK elevation was noted within a week after daptomycin was started [6]. Our patient had a significant elevation in CK 10 days after daptomycin was initiated, which is compatible with the time course of the previous cases. Among those cases, only one case report noted hyperkalemia as an early sign of rhabdomyolysis [4]. Thus, to our knowledge, this is the second case of daptomycin-related rhabdomyolysis manifesting as hyperkalemia. While hyperkalemia, in this case, could be secondary to AKI, the mechanism of hyperkalemia related to daptomycin would be explained by the cellular lysis mechanism hypothesized above, which concurrently causes hyperkalemia due to the efflux of intracellular potassium. The risk factors of rhabdomyolysis during daptomycin use were noted as concurrent statin use, obesity, diabetes mellitus, CKD, and sepsis [3]. While our patient had been on high-intensity lipophilic statins, a notorious myotoxic agent, given that rhabdomyolysis occurred 10 days after the beginning of daptomycin therapy, daptomycin is considered to be more responsible for rhabdomyolysis in our patient. Theoretically, as noted in the FDA drug label information, statins need to be held if at all possible during daptomycin administration to avoid synergistic effects of the medications, which cause rhabdomyolysis. However, our patient had a recent history of right toe amputation due to osteomyelitis and the presumption of peripheral artery disease, which increased the necessity of a statin. In cases with concurrent statin and daptomycin use, close outpatient follow-up with serial CK and renal function tests is warranted to mitigate the risk of rhabdomyolysis. While weekly CK monitoring is recommended according to the manufacturer [7], more frequent monitoring may be necessary for those with concurrent statin use. Recently, antimicrobial resistance (AMR) and stewardship have been noted as urgent global health issues [8]. Despite daptomycin's clinical usefulness, cases of daptomycin-resistant MRSA have been described in the literature. A clinical decision to use daptomycin should weigh the advantages of its convenience (once-daily injection, which would be an advantage for patients on long-term outpatient antibiotics) and its effectiveness against the risk of AMR and drug-related rhabdomyolysis.

## Conclusions

Hyperkalemia could manifest as the initial presentation of daptomycin-related rhabdomyolysis. In patients on statins, daptomycin may need to be avoided, or high-intensity statins should be held to prevent rhabdomyolysis. A decision to use daptomycin should weigh the advantages of its clinical usefulness and effectiveness against antimicrobial stewardship and the risk of drug-related rhabdomyolysis whenever possible.
